# Arrow of Time in Quantum Mechanics and Set Theory

**DOI:** 10.3390/e27090904

**Published:** 2025-08-26

**Authors:** Jerzy Król

**Affiliations:** Chair of Cognitive Science and Mathematical Modelling, University of Information Technology and Management, ul. Sucharskiego 2, 35-225 Rzeszów, Poland; iriking@wp.pl

**Keywords:** Boolean models of ZFC, quantum mechanics, arrow of time

## Abstract

The set-theory twist of quantum mechanics uncovers forcing in axiomatic Zermelo–Fraenkel set theory as a viable tool to understand the singularities in a physical spacetime and serves as a link between the quantum and classical worlds. The random forcing explains the emergence of time in quantum mechanics on infinite-dimensional Hilbert spaces. A natural flow of randomness from fully random to deterministic and classical, as in the measurement procedure and the decoherence process, becomes responsible for the cosmological arrow of time and also for the local-coordinate time in spacetime.

## 1. Introduction

Despite the effort toward the reconciliation of two fundamental pillars of contemporary physics, general relativity (GR) and quantum mechanics (QM), the goal seems unreachable at present. There are a variety of potential reasons behind this situation; some are rather technical, some are conceptual, but nevertheless something very fundamental and unrecognizable so far seems to be involved here. The experimental data to make conclusive choices are hardly in reach; however, the non-presence of supersymmetry in the LHC data is leading the attention of researchers to yet unknown theories or guesses. We follow the line of thinking that the usual mathematical tools are probably not well suited for the recognition of the problem in its full extent, or, maybe, the known tools, customarily in use in certain fields of pure mathematics, like axiomatic set theory, should be redefined and reformulated for the needs of quantum gravity and physics. An important issue here is the role that different formal models play in the axiomatic set theory. Allowing for dynamically changing models of Zermelo–Fraenkel set theory (ZFC) provides an additional perspective, or even physical degrees of freedom, which helps to understand the problem of QG in a new way; e.g., [1].

So far, this approach appears promising and quite successful. In particular, to understand the singular regions of spacetime, which are from the outside of it, one finds models of ZFC as formally assigned to spacetime singularities; e.g., [2,3,4]. This means that the outer perspective on extremely curved and deformed spacetime requires different models of ZFC (see ref. [1] for a discussion of this issue). Or, stating it differently, the integrity of spacetime is supported by certain non-imbedding properties of the set universe *V* into itself. Even more interestingly, the mathematics involved refers to certain fundamental problems in the differential topology of low-dimensional manifolds (Poincaré conjecture in dimension 4) and in QM (Tsirelson’s conjecture) [1,4,5].

The present work pushes the approach further, and we find that the dynamics of the set models may have an important impact on the emergence of time in QM. Related to this is the emergence of time on classical cosmological scales. The latter is usually thought of as the Friedmann–Lemaître–Robertson–Walker solution of the Einstein equations (EEs) under the supposition of the homogeneity and isotropy of the universe (the cosmological principle, CP). If the CP holds, then the evolution of the universe in the EEs leads to cosmic time, which is the same when measured in all frames carried by the expansion of the universe. Thus, it seems that the CP ‘explains’ cosmic time, but still based on quantum origins one would like to call for deeper, quantum, reasons for the emergence of both the CP and cosmic time. One possible reason for the CP is the cosmological inflation assigned to the very early universe as a very rapid expansion in an extremely tiny time interval, $∼10^{-32}$ s. However, to describe this abrupt process, we refer to the time in which inflation occurs, and thus the emergence of cosmological time needs further quantum explanation. This is an objection in this paper, as is the mechanism and domain in which the quantum regime assigned to the big bang leads to the emergence of cosmic time. Moreover, certain features of the expanding universe usually attributed to inflation, such as the flatness of space, can be connected with the dynamically changing ZFC models.

Another objection addressed here is the emergence of the parameter-like local time in spacetime. We insist on the different nature of the emergence of both times, cosmological and local. The first requires a purely QM derivation, while the latter can be initiated by interaction with an environment bearing macroscopic features. Nevertheless, we found a similar mechanism in the very structure of quantum mechanics behind the emergence of both times. We base our analysis on the varying models of ZFC, following the assumption that the quantum microscale determines models of ZFC different from those assigned to the classical macroscales. However, the models and their transformations are canonically determined by the QM formalism on infinite-dimensional Hilbert spaces. We close the paper with a discussion of the results and perspectives for further research.

## 2. Key Methods and Terminology

The methods here are rooted in formal set theory and are not routinely used by physicists, although they are based on well-established mathematics and their application to physics is promising and provides a completely new perspective. To help a generally oriented reader navigate the text smoothly, we provide a guide through basic concepts. Similarly to mathematical theories, physical ones, if formally presented, are based on logic and set theory. If a theory requires strict formal set-theory arguments, we usually formulate them in the first-order ZFC axiomatic system. However, this situation has another hidden structure. This is the balance between models and the language of mathematics and relies on the fact that very rich languages (such as higher-order or higher infinite languages in infinite orders) make strong set-theory suppositions regarding the structure of allowed symbols, and this influences the models of such theories [6]. It seems that higher-order theories make things easier in the sense that the stronger language can express more uniquely the intended (standard) model of the theory. However, there is a price to be paid: instead of avoiding models’ complications, we shift some difficulties of set theory into the structure of the language. The optimal balance between even first- and second-order theories is not that obvious [7]. This model/language uncertainty could also be relevant in explaining the physical world in which we live and leads to the necessity to respect a wider range of models of formal theories. This paper follows this line. In general, models are based on structures that are sets *M* or proper classes *V* in which all theorems of a theory are true (can be proved). Any consistent theory with a countable first-order language (the quantifiers range over the elements rather than subsets of a domain) has models that are sets of arbitrary infinite cardinality. ZFC is such a theory, where the language contains one binary relation ∈. We focus here on models that are standard (∈ relation is the true ‘being element’ relation), countable, and transitive, like *M*; or a proper class *V*, which is the Von Neumann cumulative universe; or are uncountable Boolean-valued models $V^{B}$, where *B* is a complete Boolean algebra in *V* and special emphasis is given to the changes between models of a given cardinality. So, given a countable model of ZFC *M*, it can be changed to a countable model, say *N*, or the class *V* to $V^{\prime }$ or $V^{B}$ to $V^{B^{\prime }}$. The method of changing M→N, $V\to V^{\prime }$ is the forcing relation, while the change of $V^{B}\to V^{B^{\prime }}$ is due to the homomorphism of Boolean algebras $h:B\to B^{\prime }$. Forcings are nontrivial whenever they add new sets (generic sets) to *M* (*V*) which were not in *M* or *V*. One way of describing forcing is via Boolean models $M^{B}$ or $V^{B}$. The condition for forcing to be nontrivial is that the Boolean algebra *B* be complete and atomless in *M* (*V*). Then, given a generic filter *G* in *B* in *M* or *V* (a filter which nontrivially intersects all dense subset of the partial order B∖{0}), we have the nontrivial extensions $M\left[G\right]:=M^{B}/G$, $V\left[G\right]:=V^{B}/G$ [8,9]:

M⊊M[G]andV⊊V[G].

The case of the universe *V* of all sets is tricky since the extensions have Boolean value 1 (the generic *G* does not exist in *V*) [10]. We come back to this in the following sections. Every model *M* or *V* or $V^{B}$, or any other from the infinitely many possibilities, is a ZFC model, which means that all ZFC provable sentences and all axioms of ZFC are equally valid in the models. Thus, any ‘observer’ rooted in the formal ZFC cannot notice any difference just by using theorems of ZFC. For such an observer, the models *M* and M[G] are the same even though *G* is generic. The observer cannot express any difference just by the provable power of ZFC. However, models differ in their properties that are independent of the ZFC axioms. This characteristic is the archetypical property of quantum randomness [11] and is also related to the arrow of time in the present work. In fact forcing was invented to show the independence of the continuum hypothesis or axiom of choice on the axioms of ZFC and ZF, respectively. However, the relation of forcing to QM is more profound. *QM on infinite-dimensional Hilbert spaces predicts forcing extensions of V.* Thus V→V[G] is a part of QM formalism. This also serves as the basis for the mechanism of the emergence of the arrow of time in QM. Note that Paul Benioff presented an early discussion of forcing and the phenomenon of changing ZFC models in QM [12,13]. To notice the forcing extension as a part of the QM formalism, we need the ZFC twist of QM on infinite-dimensional Hilbert spaces. This relies on the switch between the logic of QM on the lattice of projections and its family of Boolean subalgebras *B*s into the Boolean models $V^{B}$s. The models support nontrivial forcings V[G]s. This is explained more precisely in the next sections where suitable references from the literature are given. The important part of the QM formalism is the measurement process and the relation to observers and observations. Here we have structured QM by dependence on ZFC models and their changes, but also the observers are model dependent. In general, the state of the universe, which is an entangled state of the environment and the system, can also be seen as the entangled state of the *V*-observer and the system. *V*-observer $O_{V}$ performs the measurement procedure as if its basic set-theory model of reference is *V*. The global state of the universe is in $V^{B}$; therefore, the *V* observations project $\Psi_{V^{B}}$ into $\phi_{O_{V}}$ and $V^{B}$ to *V* but this, according to QM (ZFC twisted), is via forcing $V^{B}/G≃V\left[G\right]$. Thus, this leads to another breaking of the global symmetry of $\Psi_{V^{B}}$ [14] and to the emergence of time and its arrow in *V* for $O_{V}$. The trigger is QM not the interaction with the environment.

The way to the 2-valued universe of sets *V* via ZFC models also has its geometric or smooth topological component. Changing of the ZFC models leads to the geometry of certain smooth exotic $R^{4}$s [2]. Their exoticness means, in particular, that they are never flat $R^{4}$ (no diffeomorphism can turn them to flat smooth $R^{4}$), and hence always contain some minimal amount of the curvature density, and thus a form of gravitational energy density. We discuss this phenomenon in the following sections in the context of emerging time in QM.

## 3. The Emergence of the Parameter-like Local Time in QM

A natural way to look for the origins of time in the quantum regime is to consider the time-independent Schrödinger equation (TISE) and then show that it is transferred into the time-dependent Schrödinger equation (TDSE) of the system. This can be seen as the result of the interaction of the quantum system with the semiclassical environment, which results in the decoupling of the system and the dependence on time as a quantity that is relational to the environment [15,16,17,18]. The entire process can be based on some fundamental principle, such as symmetry breaking, which is responsible for the emergence of time [14].

In this section, we allow for another formal possibility; that is, the global entangled state Ψ leads to the emergence of time due to the above symmetry breaking, but the expressions involved are assigned to their characteristic proper models of the axiomatic set theory ZFC. Thus, there is an internal variation in the quantities due to the variation in ZFC models. The variation determines the arrow of time even though time is decoupled by the interactions with a sufficiently large semiclassical environment. The assignment of ZFC models to physical constructions or theories is a general strategy that has an impact on constructions [19]. In the next section, we show that the dynamics of ZFC models is naturally predicted by the QM formalism and that the emergence of time is also possible in QM without reference to any environment.

In some places, we are using the basic terminology of set theory as in [8]. Thus, we say that a quantum system is assigned to a model *M* of set theory when *M* is the basic formal environment in which to perform the operations and constructions of set theory needed to define the system. Let a quantum system $S_{V}$ be assigned to the ZFC model *V* (Von Neumann cumulative set universe [8]) ($S_{M}$ means that the basic model of reference is some other *M*). Similarly, the vector of state $\psi_{V}$, $\psi_{M}$ and the observer $O_{V}$, $O_{M}$ depend on V,M correspondingly as their basic set-theory models of reference. The observables P,Q,A,… are also assigned to *V* or *M*. The question arises whether all these ingredients of the description of the quantum system in spacetime are model dependent with the same phase; i.e., if the reference model changes, does it mean that it changes in the same way for all the ingredients? Thus, a constant model of reference would be distinguished at any moment (which is usually the case). Or maybe the models can differ for different constructions within a theory. We follow this second possibility.

Let the observer and the corresponding measurement procedure be defined with respect to *V* (we say ‘in *V*’). Then, given the Hamiltonian $H_{V}$ in *V* and switching it to $H_{M}$ in *M* would lead to the change $\Delta H_{V\to M}$ and, provided the observer was *V* confined, V:O, they could, in principle, observe ΔH. Thus, V:O and (V:H)→(M:H) would be the conditions to grasp the effects of the change in models.

(1) $$\begin{array}{cc} \left(V:O\right) & \to \left(M:O_{M}\right)=\left(V:O\right)\\ \left(V:H\right) & \to \left(M:H_{M}\right)\neq \left(V:H\right) \end{array}$$

which means that O is constantly based on *V*, while *H* can differ depending on the model. Writing $\left(M:H_{M}\right)\neq \left(V:H\right)$, we mean $H_{M}\neq H\left(=H_{V}\right)$ with additional information on the assignment of the model. We claim that this asymmetry in model dependence of the basic quantum mechanical components can be considered as the reason for the emergence of the time coordinate. Let *t* be a parameter that indicates a model of ZFC in the family ${\left\{M_{t}\right\}}_{t\in R}$. Then, given the dependence as in (1) with respect to the continuous *external* parameter *t* would mean

(2) $$\frac{dH(t)}{dt}\to \frac{d\lambda (t)}{dt}\;\mathrm{or}\;\frac{d\psi (x,t)}{dt}$$

where TISE reads $H_{M}\psi_{M}=\lambda_{M}\psi_{M}$ or equivalently M:Hψ=λψ.

Now let us focus on the changes of the ZFC models given by the forcing procedure; that is, we need a complete Boolean algebra $B_{V}$ in *V* and a generic filter $G\subset B_{V}$ in $B_{V}$ in *V*. The change of the model *V* to the extended model V[G] is the forcing extension V[G]⊃V of *V* [8,9]. Now, let us take a family G(t) of generic filters in $B_{V}$ parameterized continuously by the real parameter *t*. From the point of view of the *V*-observer, the change V→V[G(t)] would make changes in λ and ψ depending on the parameter *t*. G(t) can equivalently be a family of generic ultrafilters (with Boolean value 1) in the Boolean-valued model $V^{B}:=V^{B_{V}}$ [9] and $B:=B_{V}$. In the next section, we see that such a scenario of changing the ZFC models by random forcing is indeed predicted by the QM formalism on infinite-dimensional Hilbert spaces.

The usual attitude is to refer to the constant model *V* when performing the formalization of certain physical or mathematical theories, but also in this case of *V* one can extend *V* to the ‘bigger’ model V[G] with the help of the generic filter *G* in *V*. Although there is a theorem that states that generic ultrafilters do not exist in *V*, they exist with the Boolean value 1 in the Boolean model $V^{B}$. Thus, if the model $V^{B}$ is taken as the basis for the ZFC constructions, then the generic filters in *V* have counterparts in $V^{B}$ and the extension of *V*, V[G], exists with Boolean value 1 and V⊂V[G] [10].

Moreover, the approach to forcing by Boolean models is equivalent to that by countable transitive models *M* of ZFC (CTM), where the existence of generic ultrafilters in *M* and the extensions M[G] are theorems [8,9,10]. Then, an important conclusion follows: *Given a CTM M of ZFC and a complete atomless Boolean algebra B in M, there exists a continuum infinitely large family of M-generic ultrafilters ${\left\{G_{t}\right\}}_{t\in R}$ and the corresponding continuous family of forcing extensions ${\left\{M\left[G_{t}\right]\right\}}_{t\in R}$ with the property $M⊊M[G_{t}]$ for all t∈R*. Thus, there is room to consider infinitely continuous large families of forcing extensions of *M* or *V*, leading to the possible families of Hamiltonians H(t) or λ(t),|ψ(t)〉. The perfect solution to the emergence of time in the QM in the presence of the interactions with the environment would be the following dependence:

$$t\mapsto |\phi_{S}\left(t\right)\rangle =e^{-itH_{S}}|\phi_{0}\rangle$$

where $|\phi_{0}\rangle$ is the initial state of the system at t=0, $t\in R_{\geq 0}$. In fact, this can be achieved by considering the symmetry issue, following [14] and referring to the forcing properties [20]. Let |Ψ〉 be a time-independent global state of the entangled state of the system and environment. Then, the projection of |Ψ〉 onto the environment state $|\phi_{E}\rangle$ leads to singling out the system state

(3) $$\langle \phi_{S}|\langle \phi_{E}|\Psi \rangle$$

while inserting the interaction part of the Hamiltonian, $V_{I}$, decomposes the global Hamiltonian *H* as follows:

$$H=H_{S}⊗1_{E}+1_{S}⊗H_{E}+V_{I}.$$

Now, let $V^{B}:\Psi$; $V:\phi_{E}$; $V:\phi_{S}$ (we use ϕ and |ϕ〉 interchangeably). The projection (3) should be complemented by

(4) $$\begin{array}{c} \langle \phi_{S}|:=\langle \phi_{E}|\Psi \rangle \\ V≃V\leftarrow V^{B} \end{array}$$

Given a *V*-observer and the relative variation of ZFC models, it remains to demonstrate that the family $V[G_{t}]$ of the extensions of *V* leads to the timelike flow. First, our observation is that if models do not matter, i.e., if the observer, Hilbert space, observables, measurements, and Hamiltonians are not model-sensitive, or are sensitive without any relative phase between any of them, then the effect of the timelike flow would not be observed. Second, there is a decohering action of the environment on the Boolean global state $\Psi \in V^{B}$ that results in a *V*-state accessible to the observer. This is the random forcing extension V[G] in the Boolean domain that has been assigned to the ‘generic measurements’ in QM [3,19] and which extends quantum decoherence to classical decoherence. Thus, the result of environmental decoherence is not only the reduction of Ψ into $\phi_{S}$ and $\phi_{E}$ augmented by the change in the suitable ZFC models $V^{B}\to V$, but also the forcing extension $V^{B}/G≃V\left[G\right]$. Third, the nontriviality of the extension, i.e., V⊊V[G], is the generator of the time flow in QM. The precise explanation of this is given in the next section based on Refs. [2,3,19] and in the context of inflation; now, let us turn to the change in ZFC models as nested in QM and their relation to the arrow of time.

The discussion above shows that a continuously parameterized family of forcing extensions ${\left\{V\left[G_{t}\right]\right\}}_{t\in R}$ has an external linear order of its parameter *t*; however, we cannot just take this order as defining the time flow; we should rather find the internal reasons explaining the order. In other words, given two extensions $V\left[G_{t_{1}}\right],V\left[G_{t_{2}}\right]$, they are not ordered in general; however, if we allow for the subsequent forcing extensions $V\left[G_{t_{1}}\right]\to V\left[G_{t_{1}}\right]\left[G_{t_{2}}\right]\to \ldots$ and use the property

(5) $$V\left[G_{t_{1}}\right]⊊V\left[G_{t_{1}}\right]\left[G_{t_{2}}\right]⊊V\left[G_{t_{1}}\right]\left[G_{t_{2}}\right]\left[G_{t_{3}}\right]⊊\ldots$$

we obtain the linear order of the countably many forcing extensions of *V* [20,21]. Moreover, in general, it holds that

$$V\left[G_{t_{1}}\right]\left[G_{t_{2}}\right]\neq V\left[G_{t_{2}}\right]\left[G_{t_{1}}\right]$$

since $G_{t_{2}}$ is $V[G_{t_{1}}]$-generic and not *V*-generic ($G_{t_{2}}$ is the generic filter in *B* in $V[G_{t_{1}}]$ and cannot be any *B* filter in *V*). If both $V[G_{t_{1}}]$ and $V[G_{t_{2}}]$ are generic filters in *V*, then the commutativity $V\left[G_{t_{1}}\right]\left[G_{t_{2}}\right]=V\left[G_{t_{2}}\right]\left[G_{t_{1}}\right]$ holds since then the iterated forcing $V\left[G_{t_{1}}\right]\left[G_{t_{2}}\right]$ is the product forcing. More precisely, for a CTM *M*, let $B_{1},B_{2}$ be two Boolean algebras in *M* and $P\subset B_{1},Q\subset B_{2}$ two partial orders completed to $B_{1}$ and $B_{2}$ correspondingly in *M*. Then the following are equivalent ([22], Lemma 3.3):

(6) $$\begin{array}{cc} 1.\; & G\;\mathrm{is}\;\mathrm{generic}\;\mathrm{for}\;B_{1}\;\mathrm{in}\;M\;\mathrm{and}\;H\;\mathrm{is}\;\mathrm{generic}\;\mathrm{for}\;B_{2}\;\mathrm{in}\;M[G];\\ 2.\; & H\;\mathrm{is}\;\mathrm{generic}\;\mathrm{for}\;B_{2}\;\mathrm{in}\;M\;\mathrm{and}\;G\;\mathrm{is}\;\mathrm{generic}\;\mathrm{for}\;B_{1}\;\mathrm{in}\;M[H];\\ 3.\; & M[G][H]=M[H][G]=M[G\times H]. \end{array}$$

In the case of M=V we take *G* and *H* generic filters in $B_{1}$, $B_{2}$ in *V* with the Boolean value 1, which allows to perform nontrivial forcing extensions of *V* with the Boolean value 1 [10]. As we already noted above, in the general case, the extensions cannot be interchanged, which leads to the ZFC-non-commutativity of the extensions. Thus, a physical interpretation of the noncommuting observables follows. *A V-based observer, $O_{V}$, cannot simultaneously measure certain observables in V and V[G]; first a generic measurement has to be performed in V, leading to V[G], and then it is possible to perform a generic measurement in V[G] leading to V[G][H].* Note that due to the relations [10,19]

(7) $$\begin{array}{c} V\subset V^{B}\subset V\subset V^{B}\ldots \end{array}$$

(8) $$\begin{array}{c} V^{B_{1}}/G≃V\left[G\right]\;\mathrm{and}\;V^{B_{2}}/H≃V\left[H\right] \end{array}$$

it follows that the forcing extensions can be traced in *V*, leading to the known uncertainty relations for noncommuting observables envisaged by $O_{V}$. For now, we leave this potentially fundamental role of the ZFC asymmetry in QM and focus on the emergence of the time parameter driven by this.

Similarly to reshaping the spacetime in the initial epoch of universe, when inflation causes superluminal expansion, the changing models of ZFC as in the forcing extension are out of the spacetime rigor. However, both can have observable indirect consequences. We connect physical time with the process of changing the ZFC models; this process is not necessarily unitary and is outside of the spacetime causality. Let $\Theta_{t_{1},t_{2}}\Theta_{1,2}$ be the operator of forcing extension $\Theta_{1,2}:V\left[G_{1}\right]\to V\left[G_{1}\right]\left[G_{2}\right]$ for generic filters $G_{1},G_{2}\subset B$ in *V*, where *B* is a complete Boolean algebra and $\Theta_{0,1}:V\to V\left[G_{1}\right]$. Let ${\left\{G_{t}\right\}}_{t\in R_{\geq 0}}$ be a family of generic filters in *B* directed by the forcing extensions governed by the operator $\Theta_{i,j}$ as follows.

For every countable family ${\left\{G_{i}\right\}}_{i\in I\subset N}$ chosen from ${\left\{G_{t}\right\}}_{t\in R_{\geq 0}}$ of the generic filters in *B*, it holds that

$$\Theta_{i_{1},i_{2}}:V\left[G_{i_{1}}\right]\to V\left[G_{i_{1}}\right]\left[G_{i_{2}}\right]\;\mathrm{for}\;\mathrm{any}\;i_{1}<i_{2}\in I.$$

The parameter $t\in R_{\geq 0}$ can be identified with the time parameter. First, we see that there is linear order on the space of values of *t*, which reflects the ordering of [0,+∞) but results from the forcing extensions. Second, given $t_{1}<t_{2}$ or $\Delta t_{1,2}$, one can assign to it the change in the curvature of the local 4-dimensional Euclidean smooth manifold underlying a Lorentzian spacetime. This change in curvature is connected with the change in gravitational energy, though the latter is not covariantly defined as a local quantity in spacetime. The assignment should follow the uncertainty relation $\Delta t_{1,2}\cdot \Delta E_{curv,1,2}>\hbar$ to be observed. Third, the forcing-based parameter *t* helps to understand the transition of TISE to TDSE for quantum systems on infinite-dimensional Hilbert spaces $H^{\infty }$. More precisely, the entangled global state Ψ (of the environment and the quantum system) lives in $H^{\infty }$, while the projected part of the system can be finite-dimensional: $\phi_{S}\in H_{S},H_{S}^{\infty }$. The projected part of the environment is expected to be infinite-dimensional: $\phi_{E}\in H_{E}^{\infty }$.

The entanglement of Ψ is reduced by projection to the Cartesian product of $\phi_{S}$ and $\phi_{E}$ but the formalism of extensions of ZFC models adds subtly to the issue of reduction. That is, according to (4), the decoupling of $\phi_{E}$ and $\phi_{S}$ from Ψ is accompanied by a $V^{B}\to V$ process in the models, and as long as this results in *V* it is fully reduced. However, there is an incomplete reduction to $V^{B}/G_{i}≃V\left[G_{i}\right]$ properly extending the set universe *V* (see (8)). This is the generator of the time shift $\Theta_{0,i}:V\to V\left[G_{i}\right]$. The next step would be $\Theta_{i,j}:V\left[G_{i}\right]\to V\left[G_{i}\right]\left[G_{j}\right]$. The relation (4) should be modified to

(9) $$\begin{array}{c} \langle \phi_{S}|:=\langle \phi_{E}|\Psi \rangle \\ V\subset V\left[G\right]\leftarrow V^{B}. \end{array}$$

which can be extended to the double-iterated forcing (and further to the multiply iterated forcing):

(10) $$\begin{array}{cc} \; & \langle \phi_{S}|:=\langle \phi_{E}|\Psi \rangle \\ V\subset & V\left[G_{i}\right]\subset V\left[G_{i}\right]\left[G_{i+1}\right]\leftarrow V^{B}\\ \; & ↑\\ \; & V^{B}. \end{array}$$

The meaning of this is as follows: The remnant Boolean entanglement V[G] generates the arrow of time in QM. This means that given a formalism of QM in a single model *V*, this is symmetric and the arrow of time does not appear. These are changes of ZFC models that break the symmetry and lead to the arrow of time. From the point of view of the *V* observers, there is no observed V[G] (no generic filter *G* exists in *V*), nevertheless the arrow of time emerges as an observed quantity fulfilling the uncertainty relation with energy.

To have a directed family of iterated forcing extensions, one cannot collapse to *V* at any step of the iterations (since then the linear order of extensions (5) would be broken). Thus, the remnant, i.e., not reduced to *V*, Boolean entanglement $V\left[G_{i_{1}}\right]\ldots \left[G_{i_{k}}\right]$ must be present at every step *k*.

This is in fact the tension between two different perspectives in set theory, one based on the distinguished unique model *V* and the other where set-theory multiverses become fundamental. This second perspective means, in particular, that forcing is primary and each model would be a forcing extension of the other ground model. In the present context, the forcing perspective follows the families of extensions from $V^{B}$ (multiverse perspective), while the *V*-perspective is assigned to the *V*-based observer. Roughly, the observer based on *V* and the QM world following the forcing extensions (multiverse) give rise to the emergence of classical time. Before showing this more precisely, let us find the reasons for preserving the Boolean entanglement rather than collapsing into the 2-valued classical world *V*. We turn to the uncertainty of time and energy; in a very short time, we can have an indeterminacy of energy following the uncertainty QM relation. Let us consider a scenario where spacetime expands and the matter content remains unchanged. That would mean that the density of matter or energy would decrease. However, when the expansion takes place in a very short time, the small change in the energy density could be compensated in an unobserved way by a suitable, smaller than required by the ΔE·Δt<ℏ, bound. This is quite analogous to the creation and annihilation of virtual particles carrying the interactions in quantum field theory. The particles are unobserved (off-shell) as far as they are created and annihilated in a short time, negating the uncertainty relation with respect to the masses/energies of the particles. However, their virtual existence gives rise to observable physical effects. Here, the expansion is due to the internal extension of the real ax $R_{V}$ to $R_{V[G]}$ and compensation of the densities of energy virtually in a suitably small time interval, and finally ending at an expanded universe built on $R_{V[G]}^{4}$ with the compensated densities of energy. Thus, the amount of increase in the energies ΔE and the time interval Δt fulfill ΔE·Δt<ℏ, which for very small time intervals gives rise to a considerable compensation in energy, leaving the final state of spacetime in V[G], and so on. This process of expansion due to forcing and compensating energies (due to the uncertainty relation) refers, however, to the coordinate time, which we show how to derive. To speak about uncertainty time/energy at the single forcing extension step, we need to have a notion of time to refer to.

Following [14], the TISE is written in the fully symmetric form for the global entangled state Ψ=|Ψ〉:

(11) $$e^{i\lambda (\bar{H}-E)}\Psi =\Psi ,\;\lambda \in C.$$

The derivation of this expression with respect to λ leads to TISE $(\bar{H}-E)\Psi =0$. It is sufficient to consider the real parameter t=ℏλ. As shown in [14], breaking the symmetry of (11) due to the decoupling of the system and environment states from Ψ (the projection (4)) leads, in a sequence of transparent steps, to TDSE for the system states $\phi_{S}\left(\lambda \right)$

(12) $$\left({\bar{H}}_{S}+V_{S}\left(\lambda \right)\right)\phi_{S}\left(\lambda \right)=i\frac{d}{d\lambda }\phi_{S}\left(\lambda \right)$$

Taking λ=t/ℏ, we retrieve the TDSE for a quantum system where the time-dependence emerges due to the interaction of the global state with the environment. We claim the arrow of time, that is, the directed reparameterization

(13) $$Re\;\lambda \left(s\right)=t^{\to }\left(s\right):=t\left(s\right)/\hbar$$

is generated by the underlying forcing extension, as in (9).

In general, λ(s) and the expressions where it appears are parameter *s*-independent [14], so there is no clear reason for choosing this or another parameterization of λ as the function of *s* in the fixed ZFC model like *V*. Let $V:\lambda \left(s\right)=\lambda {\left(s\right)}_{V}$ in *V* be parameter-independent, and let $V\left[G\right]:\lambda \left(s\right)=\lambda {\left(s\right)}_{V[G]}$ in V[G] also be parameter-independent. There is, however, $\Delta t_{V\to V[G]}$ due to the unobserved off-shell breaking the uncertainty time–energy bound, where the change of models generates the change in energy (see the next section). Then, the observed ordering of *t* results for ΔE·Δt>ℏ. This is the order given by V⊂V[G] that becomes a part of the *t* flow such that

(14) $$\begin{array}{c} t_{i}\in V,t_{j}\in V\left[G\right]\;\mathrm{and}\;t_{i}<t_{j}\;\mathrm{within}\;a\;\mathrm{tiny}\;\mathrm{interval}\;\Delta t\;\mathrm{given}\;\mathrm{by}\;\Delta t\cdot \Delta E<\hbar \\ \mathrm{for}\;\mathrm{any}\;\mathrm{parametrization}\;t(s). \end{array}$$

This is a virtual origin of the arrow of time based on set-theory forcing, as shown in the next section and which can be called the quantum origin of the local arrow of time. The arrow of time and time are deep quantum properties that transcend the spacetime regime.

## 4. The Emergence of Cosmological Time and Inflation

Forcing has been considered so far as an external possibility for QM rather than an inherent part of the QM formalism. In addition, the energy appearing in ΔEΔt is given in very general terms, missing the relation either to QM or to forcing. We want to fill this gap and first perform a certain reformulation of the QM formalism, uncovering the close connection with set theory. This is the ZFC twist of QM that has been found and described in earlier work, e.g., [19]. Secondly, we build an interpretation of *E* that is closely connected with the curvature of 4-spacetime; e.g., [1].

The issue of the emergence of time at the very early stages of the universe, say during inflation, is something that seemingly creates a vicious circle. To define the expansion of universe, we need time, which should be presumably defined by the expanding universe itself. However, inflation is not a process in spacetime, it is the process of transforming spacetime which is not directly observed in spacetime; nevertheless, this has observational indirect effects. An observation like that is not any solution to the conundrum of the emergence of the arrow of time, but is a valid indication toward it. The superluminal extension of spacetime and the extension of ZFC models by forcing as underlying the dynamics of spacetime at the initial stages of the universe’s evolution can be considered as inherently related. However, here the dynamics of ZFC models is placed on a more fundamental level, which can formally explain the emergence of time and the effect of inflation.

In the previous section we found a special role for set-theory forcing in the emergence of the arrow of the local parameter time. Now, we want to show that forcing is also a useful technique for understanding the physical process of inflation and the emergence of cosmological time. Thus, the vicious circle would be escaped by the observation that time and the expansion of the universe can be seen as quantities derived from the dynamics of set-theory models and do not necessarily depend on each other. This requires a deeper rooting of the forcing in the QM formalism.

Suppose that the initial phase of the quantum universe is described by the state Ψ in H, dimH=∞. The logic of QM is typically described by the lattice of projections L(H)=(L,0,1,∧,∨), where the infimum operation ∧ of a family of projections $\left\{P_{\alpha }\right\}$, $\wedge \{P_{\alpha }\}$, is the projection on the intersection of all ranges of $P_{\alpha }$, and the supremum $\vee \{P_{\alpha }\}$ is the projection on the complementation of the sum of all ranges of $P_{\alpha }$. This lattice is never distributive unless dimH=1. Let $B_{max}$ be a complete maximal Boolean algebra of projections chosen from L, $B_{max}\subset L$. It represents a maximal Boolean context in QM that is certainly distributive. In the particular case of B={0,1}=2, of the 2-valued Boolean algebra, the context is 2-valued and the logic is classical. This logical side of QM given by L has been widely acknowledged and elaborated since the original formulation by Von Neumann and Birgkhoff [23].

The path to the set-theory side of QM, as complementary to the logical one, is not that unique and clear. However, for infinite-dimensional Hilbert spaces the picture becomes tame and contains universal ingredients. The core of the switch between logic and set theory is the following relation:

$$\mathrm{Complete}\;\mathrm{Boolean}\;\mathrm{algebra}\;B\;\mathrm{of}\;\mathrm{projections}\;\mathrm{in}\;L⟷\;\mathrm{Boolean}\text{-}\mathrm{valued}\;\mathrm{universe}\;V^{B}\;\mathrm{of}\;\mathrm{sets}.$$

The point is that $V^{B}$ is the (Boolean-valued) model of ZFC and *B* is canonically determined by QM on infinite-dimensional Hilbert spaces. On the other hand, *B* comprises the values of the spectral families of all pairwise commuting self-adjoint operators (we say in *B*). This is yet another feature of the Boolean local context *B* of QM. The rationale behind the reference to the ZFC twist of QM on infinite-dimensional Hilbert spaces is the following.

**Lemma** **1.** *If dimH=∞ and $B_{max}\subset L\left(H\right)$, then $B_{max}=B⊕B_{a}$, where B is the atomless complete Boolean algebra that is the same for all $B_{max}\subset L$ while $B_{a}$ is the atomic summand. $B_{a}$ varies depending on $B_{max}$.*

**Corollary** **1.** *B=Bor(R)/N of Borel subsets of R modulo the ideal of Lebesgue-measure-zero subsets. B is the same for every $B_{max}\subset L\left(H^{\infty }\right)$.*

In other words, *B* is the Boolean measure algebra on R and $V^{B}$ is the canonical Boolean model of random Solovay forcing [24]. However,

**Corollary** **2.** *If dim(H)<∞, then $B_{max}=B_{a}$.*

This means that the set-theory side of the QM formalism on $H^{\infty }$ is universally affiliated with the change of ZFC models due to random forcing. This random forcing, that is,

$$V^{B}/G≃V\left[G\right],\mathrm{where}\;G\subset B\;\mathrm{is}\;a\;\mathrm{generic}\;\mathrm{filter}\;\mathrm{on}\;B=Bor(R)/N\;$$

is at the root of the local arrow of time, but also helps to understand cosmological time. In the usual logical approach to QM, that is, by the lattice of projections on $H^{\infty }$, this property of dynamical ZFC models is completely hidden, and only becomes transparent after performing the ZFC twist of QM.

Now, given a directed family of random forcing extensions of *V*, as in (5) and (6), where B=Bor(R)/N is the same at every step, we can formulate the condition for the family to be described by the sequence of consecutive forcing extensions. This means that we do not want to allow the 2-valued reduction to be *V*, rather we have

(15) $$\ldots V{\left[G_{i_{1}}\right]}^{i_{1}}:=V\left[G_{1}\right]\left[G_{2}\right]\ldots \left[G_{i_{1}}\right]\to \ldots V{\left[G_{i_{1}+1}\right]}^{i_{1}+1}$$

at every step, $i_{1}\to i_{1}+1$. That is, each $G_{j},j=1,\ldots ,i_{1}+1$ exists in $V^{B}$ with Boolean value 1. Equivalently, the sequence (1) contains only nontrivial forcing extensions at each step.

The QM formalism on infinite-dimensional Hilbert spaces leads to the change of the ZFC models like V→V[G]; thus, even though the initial global state was $\Psi_{V}$, there emerges $\Psi_{V[G]}$. Provided we have a notion of the energy assigned to the change of ZFC models, this change generates the time flow in the off-shell mode according to ΔE·Δt<ℏ. From the point of view of the alleged *V*-observer at the very early universe, any observation cannot show the birth of the arrow of time, but rather time is a primordial quantum phenomenon. The Boolean model $V^{B}$ is reduced to *V*, $V^{B}\to V$, according to ΔE·Δt<ℏ, which assumes the nontriviality of the forcing extension $V[G_{i}]$, that is, $V^{B}\to V^{B}/G_{i}≃V\left[G_{i}\right]$. Although $V[G_{i}]$ is not observed as an extension of *V*, any other extension already starts with $V[G_{i}]$, that is, $V\left[G_{i}\right]\to V\left[G_{i+1}\right]$, etc. This process is the basis for the arrow of time in the very early universe, where it is difficult to assume the action of any external environment, as was the case with the local parameter time in the previous section.

Thus, the above scenario depends on the notion of energy assigned to forcing extensions and to the emergence of spacetime from the primordial global state Ψ. A suitable energy would be the gravitational energy density, but facing a difficulty with the covariant formulation of the gravitational energy, one refers to the density of 4-curvature in certain regions of spacetime [1]. We follow two basic assumptions; first, spacetime in extreme conditions can be fragmented and lose its integrity such that the remnants of this process are (i) flat objects, $R^{4}$s in $V^{B}$s; and (ii) the family of relations between flat $R^{4}$s. If initially spacetime was curved or contained a nonvanishing curvature density (gravitational energy density), this energy must be stored after fragmentation in the relations between flat $R^{4}$s [1]. So, after eventual reconstruction of the spacetime manifold in a single-ZFC model, say *V*, this curvature density would be retrieved.

The reverse process of defragmentation of the spacetime region starts with flat $R^{4}$s, which, after patching together, lead to a 4-dimensional spacetime and the energy density becomes a part of the spacetime description. The retrieval of 4-spacetime back from the flat ${\left\{R_{\alpha }^{4}\right\}}_{\alpha \in I}$ in $V_{\alpha }^{B}$s and the family of relations $R_{\alpha \beta }:R_{\alpha }^{4}\to R_{\beta }^{4}$ as a smooth 4-manifold in the single model *V* is called the *V*-smooth limit of fragmented spacetime. The basic property of the fragmentation followed by the defragmentation processes is the following [1].

Exotic $S^{4}$, only theoretically postulated so far, are smooth 4-spheres which are homeomorphic but not diffeomorphic to the standard smooth $S^{4}$, that is,

$$\mathrm{Exotic}\;S^{4}\overset{diff.}{≄}\mathrm{standard}\;S^{4};\;\mathrm{exotic}\;S^{4}\overset{homeom.}{≃}\mathrm{standard}\;S^{4}$$

Similarly, with exotic smooth $R^{4}$,

$$\mathrm{Exotic}\;R^{4}\overset{diff.}{≄}\mathrm{standard}\;R^{4};\;\mathrm{exotic}\;R^{4}\overset{homeom.}{≃}\mathrm{standard}\;R^{4}$$

But continuum infinitely many such $R^{4}$s [25] have been found by mathematicians. The existence of an exotic $S^{4}$ would contradict the celebrated smooth 4-dimensional Poincaré conjecture [26].

**Theorem** **1** ([1], Theorem 4; [2])**.** *Suppose that exotic $S^{4}$s exist. Then the V-smooth limit of fragmented $S^{4}$ has to be an exotic $S^{4}$ and the V-smooth limit of fragmented standard $R^{4}$ has to be some exotic $R^{4}$.*

Recall that $S^{4}\setminus \left\{pt.\right\}≃R^{4}$ and in the case of exotic $S^{4}$ this $R^{4}$ would be an exotic $R^{4}$ that has not been found so far; no known exotic $R^{4}$s support exotic $S^{4}$ in this way. In other words, any known exotic $R^{4}$ complemented by a point in infinity gives rise to the necessarily standard smooth $S^{4}$. The conclusion of Theorem 1 is the following.

**Corollary** **3.** *The V-smooth limit of the flat fragmented $R^{4}$ is exotic $R^{4}$, supporting the existence of exotic $S^{4}$ or being one of the known exotic $R^{4}$ (which do not support any exotic $S^{4}$).*

The crucial property of exotic $R^{4}$s as modeling the region of spacetime is the following [3,25]: *Any exotic $R^{4}$ cannot have the vanishing Riemannian tensor, hence the vanishing globally Riemannian 4-curvature* (since then, it would be diffeomorphic to the standard $R^{4}$).

Now we have sufficient tools to approach the energy ΔE issue accompanying the forcing extensions in spacetime. Theorem 1 indicates that the fragmented, even flat, region of spacetime in the *V*-smooth limit, after the defragmentation process in the classical 2-valued environment *V*, becomes exotic smooth $R^{4}$. This means that there has to appear nonvanishing curvature density, or the gravitational energy density, according to the above stated property of exotic $R^{4}$s. This growth in energy density, if coupled with Δt violating the uncertainty time–energy relation, is unobservable, and as a result this virtual ΔE generates the Δt prior to any observation. However, let us consider the rapid energy multiplication of this process of virtual change in energy such that global uncertainty $\Delta E_{mult.}$ and Δt fulfill the uncertainty relation $\Delta E_{mult.}\cdot \Delta t>\hbar$. These can be observed and lead to the emergence of cosmological time as a physical on-shell quantity. Its direction agrees with the multiple forcing extensions of the ZFC models (1).

The above constructions assume that there is a qualitative and quantitative sense attributed to time and energy even before spacetime has emerged from the purely quantum regime. This is not surprising if one thinks of the quantum realm as being more fundamental than classical. Also, an observer is connected to spacetime, and this is the regime where the uncertainty relation of time and energy gives the bound for the measurements. Thus, the emergence of spacetime from Ψ and the emergence of cosmic time occur in parallel, and inflation subsequently applies to spacetime as a whole.

Let us recall that cosmic time is the time experienced by an observer at rest in a frame carried by the expansion in an isotropic and homogeneous universe. So, it is the time measured by an observer’s clock remaining motionless for the homogeneous matter distribution in the universe, and hence carried along with it by the expansion. If the universe really is isotropic and homogeneous and its expansion is driven by forcing extensions of the ZFC models, all co-mobile observers should measure the same cosmic time between two events.

A commentary is in order. It seems that for the above scenario to work, one could introduce a minimal amount of time flow, like $\Delta t≃T_{min}$, at the quantum level even before spacetime emerged, so that given ΔE in $V[G_{i}]$, the corresponding $\left|\Delta t\right|\geq T_{min}$. This would require a preclassical, quantum form of ‘quantized’ time. It is known that there are problems with assigning a self-adjoint operator to the time variable, thus such quantized time could be something primordial (though, see [27]). In such a case, repeating the forcing extensions *i* with a certain $\Delta E_{i}$ at the *i*th stage, then $\Delta T_{i}$ accumulates and eventually exceeds the uncertainty bound. However, there is also the notion of continuum multiple forcing, which could lead to the exceedance property. We do not need to decide here which case is referred to, but it is quite intriguing to ask such questions.

As has been implicitly present in the description, the emergence of exotic $R^{4}$ as above is closely connected with the forcing extensions derived from QM. The details have been presented in [1] and the main points of construction are the following.

(i) The lattice of projections $L(H^{\infty })$ is approximated by a family of its maximal Boolean algebras, and each of them contains as a summand the complete maximal measure Boolean algebra *B*. Thus, the family ${\left\{B_{\alpha }\right\}}_{\alpha \in I}$ of Boolean contexts approximates the quantum lattice of projections. The important point is the observation that the lattice $L(H^{\infty })$ can never be reduced to a single *B*.
(ii) The ZFC twist of QM enables the inclusion of Boolean models of ZFC in the formalism $\left\{V^{B_{\alpha }},B_{\alpha }\in {\left\{B_{\alpha }\right\}}_{\alpha \in I}\right\}$. Each $V^{B_{\alpha }}$ hosts the flat object $R_{\alpha }^{4}$.
(iii) The remnants of the fragmented spacetime smooth manifold constitute the family of flat objects $\left\{R_{\alpha }^{4}\in V^{B_{\alpha }}\right\}$, and the family of relations $R\{R_{\alpha \beta }:V^{B_{\alpha }}\to V^{B_{\beta }}\}$ leads to the family of the relations $\left\{R_{\alpha \beta }^{\prime }:R_{\alpha }^{4}\to R_{\beta }^{4}\right\}$ between $R_{\alpha }^{4}$ objects. The family R is generated by the automorphisms of *B*, AutB.
(iv) In the *V*-smooth limit, it is shown that R reduces to the family of nonidentity diffeomorphisms of $R^{4}$, $Diff\;R^{4}$. This last property is equivalent to the exotic smoothness structure on $R^{4}$ in *V* [1].
(v) The smooth *V* limit above is augmented by the family of random forcing extensions supported by $V^{B}$ [24]. The generic measurements in QM on infinite-dimensional Hilbert spaces are always accompanied by the random forcing [3], building the link with a class of random phenomena, negating the recently proved Tsirelson’s conjecture [5,28].
(vi) The remnants of spacetime in (iii) above are considered to be the final state of the gravitational collapse leading to a black hole singularity. Flat objects $R_{\alpha }^{4}$ are the basic constituents of defragmented spacetime as a smooth manifold evolving from the global QM state Ψ.

The emergence of the arrow of time in QM, which is the main objective of this paper, comes from the random forcing, which, in turn, can be fully understood as part of the QM formalism by the ZFC twist. The latter also allows for decoupling the smooth spacetime manifold from the primordial quantum state Ψ, as signaled in (vi) above.

It remains to focus on inflation from the point of view of ZFC models. Rapid expansion of spacetime is the continuation of the uncertainty bound ΔE·Δt below *ℏ* in the quantum regime, over the on-shell observationally accessible region of expanding spacetime. To properly address this issue, we should refer to some properties of random forcing. The fairly general result deals with any forcing extension of a CTM *M*, M[G]. Let $\mu^{*}\left(A\right)$ be the outer Lebesgue measure of *A* and $\mu_{*}\left(A\right)$ the inner Lebesgue measure of *A*. Recall that whenever $\mu^{*}\left(A\right)=\mu_{*}\left(A\right)$ then *A* is Lebesgue-measurable. Let $R_{M}$ and $R_{M[G]}$ be real lines in *M* and M[G] correspondingly.

**Lemma** **2** ([29])**.** *1. $R_{M}\subset R_{M[G]}\subset R$.*

*2.*  *$R_{M}$ is a nonmeasurable subset of $R_{M[G]}$ in M[G].*
*3.*  Every Lebesgue-measurable subset $S\subset R_{M}$ with $\mu_{*}\left(S\right)=0$ is Lebesgue-measurable with μ(A)=0.

Noting that $\mu_{*}\left(R_{M}\right)=0$, $\mu^{*}\left(R_{M}\right)=1$ [30], we have for any measurable subset *S* of $R_{M}$,

$$\mu \left(S\right)=0,\;S\subset R_{M}\;\mathrm{in}\;M\left[G\right].$$

We want to see the forcing extension of ZFC models as generating inflation in a suitable sense; that is, forcing would be a driving force for inflation. The process M→M[G] sends $R_{M}\to R_{M[G]}$. But (M:R)→(M[G]:R), where $R_{M}$ in *M* has a full Lebesgue measure 1 in *M*, while each measurable subset $S\subset R_{M}$ in M[G]$\mu_{M[G]}\left(S\right)=0$, and there is no measurable subset of $R_{M}$ in M[G] with a Lebesgue measure larger than 0. The same applies to $R_{M}^{4}\subset R_{M[G]}^{4}$ in M[G]. This effect of taking any subset (Lebesgue-measurable) in $R^{4}$ to the set of measure 0 in M[G] resembles a rapid extension of space. We try to make the forcing between ZFC models responsible for the process of rapid expansion of spacetime, where inflation would be the derived concept. Since forcing on the CTM of ZFC can be seen as equivalent to forcing on Boolean-valued models such as $V^{B}$ [10], we can apply the above observations regarding the CTMs *M* and M[G], also for *V* and V[G].

An arbitrarily dense object $O_{V}\subset R_{V}^{4}$, represented by the measurable subset of its set of coordinates in $R^{4}$, after generic extension, is described as a zero-measure subset of $R_{V[G]}^{4}$. Intuitively, it can be represented by the process of adding continuum infinitely many new geometric points between the points of $O_{V}$. One immediate application could be the flatness problem of the observed universe in cosmology. This is routinely solved by assuming inflation [31]; however, here we can address this problem by extensions of ZFC models. Let the starting point of the evolving universe be the curved space $≃R_{V}^{3}$ in *V*, and then due to the forcing extension of the ZFC model in V[G]. This process is driven by QM, as explained earlier. Now we can turn to Lemma 2 and determine the density of energy and matter $\rho_{V}$ in $\rho_{V[G]}$. Let $\rho_{V}$ be spread in *V* over any measurable subset *S* of $R_{V}^{3}$, then $\int_{S}\rho_{V}\left(\bar{x}\right)d\bar{x}\neq 0$. In V[G] this reads

$$\int_{S_{V[G]}}\rho_{V[G]}d_{V[G]}\bar{x}=0,\;\mathrm{since}\;\mu_{V[G]}\left(S_{V[G]}\right)=0.$$

This means that the curvature of space due to the density of matter and energy in the extended model V[G] vanishes.

This change in the internal geometric density of the set R itself does not suffice, however, for modeling the inflationary rapid expansion of the universe. It still holds that $O_{V}$ is dense in $O_{V[G]}$, similarly to rational numbers that are in the countable Lesbegue-measure-zero set are dense in R. We need a more radical tool than just a single forcing extension, and we have already suggested that this work can be achieved by multiple forcing. In fact, continuum many-forcing extensions can be used, and this is by the same token responsible for the emergence of time. The proper definitions and proof of the existence and properties of the large arbitrary α family of forcing extensions can be found in [22] (Definition 4.11; Lemma 4.12). Here, α∈Ord is an arbitrary ordinal, and the corresponding family of forcing extensions ${\left\{G_{x}\right\}}_{\alpha },x\leq \alpha$ can be successfully defined. In our case, we need α=c, x≤c (see also (1) and (5)).

**Lemma** **3.** *There exists a family of continuum infinitely many multiple forcing extensions, ${\left\{V\left[G_{x}\right]\right\}}_{x\leq c}$, of V.*

Take the family ${\left\{V\left[G_{x}\right]\right\}}_{c}$ labeled by the ordinal type of real numbers R, i.e., ${\left\{V\left[G_{x}\right]\right\}}_{x\in R}$.

Let μ() be a Lebesgue measure on R and define the integration over forcing extensions as

(16) $$\int_{{\left\{V\left[G_{x}\right]\right\}}_{x\in R}}f\left(x\right)dx,\;\mathrm{where}\;f\left(x\right)\in V\left[G_{x}\right]$$

Now take $x=G_{x}$, which means that *x* is a generic real in $V[G_{x}]$ such that the generic filter that generates this real is $G_{x}$. We have

$$\int_{{\left\{V\left[x\right]\right\}}_{x\in R}}f\left(x\right)dx,\;x\in V\left[x\right].$$

The measure $\mu_{R}\left(\right)$ generating dx above is called a *forcing value measure on* R. Then,

(17) $$\int_{{\left\{V\left[G_{x}\right]\right\}}_{x\in R}}1\cdot dx=R.$$

That is, the integration retrieves the index set from the family ${\left\{V\left[G_{x}\right]\right\}}_{x\in R}$. If the index set were $R_{V[G]}$, then $\int_{{\left\{V\left[x\right]\right\}}_{x\in R_{\left[V[G\right]}}}1\cdot dx=R_{V[G]}.$

The point is that allowing such continuous infinite forcing extensions in a model of the evolving universe leads to the separation of points in *V* by a nonempty open interval. In the single extension V→V[G], the separation is of infinitesimal order dx—the old reals $R_{V}$ are dense in the extended case $R_{V[G]}$. In the continuous family of forcing extensions, they can be integrated out to the homeomorphic interval to R, as in (17), and this integrated interval becomes part of the extended ZFC model as a geometric real line determining the arrow of time. In this way, we arrive at the intriguing possibility that the forcing extensions of ZFC models could be considered to some degree as an alternative to the well-established inflation scenario. However, more work is needed to fully justify this possibility.

We summarize our considerations with a more unifying perspective, in particular, with regard to the presence of observers. The ZFC twist of QM starts with the lattice of projections L and the quantum set-theory universe for QM [19,32,33]:

$$L\overset{\mathrm{ZFC}\;\mathrm{twist}}{\to }V^{L}.$$

The next step is the approximation of L by the family of maximal complete Boolean algebras ${\left\{B_{\alpha }\right\}}_{\alpha \in I}$ and the family of the relations between them ${\left\{{\mathrm{Rel}}_{\alpha \beta }:B_{\alpha }\to B_{\beta }\right\}}_{\alpha ,\beta \in I}$:

$$L\to {\left\{B_{\alpha }\right\}}_{\alpha \in I}\cup {\left\{{\mathrm{Rel}}_{\alpha \beta }:B_{\alpha }\to B_{\beta }\right\}}_{\alpha ,\beta \in I}.$$

Then, applying the ZFC twist for the right-hand side gives

$${\left\{V^{B_{\alpha }}\right\}}_{\alpha \in I}\cup {\left\{h_{\alpha \beta }^{*}:V^{B_{\alpha }}\to V^{B_{\beta }}\right\}}_{\alpha ,\beta \in I}.$$

For dim(H)=∞, significant simplifications emerge [1]. First, $B_{\alpha }=B=Bor/N$ for every α∈I (Lemma 1, Corollaries 1 and 2). Second, $\left\{{\mathrm{Rel}}_{\alpha \beta }\right\}$ become automorphisms of *B*, i.e., ${\mathrm{Rel}}_{\alpha \beta }\in Aut\;B$ for any α,β∈I. Third, $\left\{h_{\alpha \beta }^{*}\right\}$ become automorphisms of the Boolean model $V^{B}$, $h_{\alpha \beta }^{*}\in Aut\;V^{B}$ [1]. Fourth, and crucial for the emergence of the arrow of time in QM, $V^{B}$ supports nontrivial random forcing, i.e., V⊊V[G].

Let us introduce the observers into the formalism. They are *V*-based (they are defined in *V* or *V* is the basic reference universe of sets), which means that potential effects from other models of ZFC could be faced by such observers. For the primordial quantum state of the universe, which is in $V^{B}$, we do not assume the existence of any semiclassical observer in $V^{B}$. However, the effects of this initial epoch can be traced back by the *V*-observers in the current state of the universe. This is not the same as the emergence of time by any interactions with a semiclassical environment; we do not assume this but it is an empirical fact that the observers in the macro-classical world exist and to refer to this is rather natural.

The cosmological global arrow of time, along with inflation effects, is due to the change in the *V* perspective and applied to the $V^{B}$ states. The point is that there exists a class of QM observations, called generic observations [19], which together with the reduction of the wave function lead canonically to the forcing extension $V^{B}/G\to V\left[G\right]$. These forcing extensions, as we have already emphasized, are predicted by the QM formalism (they follow from the ZFC twist of QM) and are parts of the QM rather than the classical environment. Or better, the models *V* and V[G], but also the dynamical change of them V→V[G], all are predicted by QM, and this last dynamics is the primary reason for the emergence of the arrow of time in QM without the interactions with any semiclassical environment. The formal derivation of the time dynamics can follow, as previously, breaking of the global symmetry (3) of the entangled state Ψ, but now it is driven by the forcing of the ZFC models. This leads to the projection of Ψ on $\phi_{OV}$ (the state of the *V*-observer) and decoupling $\phi_{S}$ (the state of the system) from the entangled Ψ, and results in TDSE. All this without interactions with the environment; that is, $H_{I}=0$. Thus, there are two mechanisms for breaking the global Ψ symmetry: one through interactions with the environment, and the other through the generic QM measurement of the *V* observer, which contains the dynamics of ZFC models and forcing extensions. Breaking of the global symmetry through forcing gives rise to the parameter, say $\lambda^{\prime }$, which is interpreted as time. Let us be more direct. The global state Ψ is projected on $\phi_{OV}$:

(18) $$\begin{array}{c} \langle \phi_{O_{V}}|\Psi \rangle \;\mathrm{such}\;\mathrm{that}\;\mathrm{the}\;\mathrm{global}\;e^{i\lambda^{\prime }\left(\hat{H}-E\right)}\Psi =\Psi \;\mathrm{which}\;\mathrm{is}\;\mathrm{equivalent}\;\mathrm{to}\;\mathrm{TISE}\;\\ \;\mathrm{gives}\;\mathrm{rise}\;\mathrm{to}\;\;\langle \phi_{O_{V}}|e^{i\lambda^{\prime }\left(\hat{H}-E\right)}\Psi . \end{array}$$

Consider the parameter $\lambda^{\prime }$ that distinguishes states in *V* (and is assigned to $O_{V}$ in $H_{V}$) as labeling the states of the system $|\phi_{S}\left(\lambda^{\prime }\right)\rangle$ as follows:

$$|\phi_{S}\left(\lambda^{\prime }\right)\rangle :=e^{i\lambda^{\prime }\left(\hat{H}-E\right)}|\phi_{S}\left(0\right)\rangle$$

for some initial value $\phi_{S}\left(0\right)$. The family of states $|\phi_{S}\left(\lambda^{\prime }\right)\rangle$ assumes the changes of the models $V^{B}\to V$, where the family of generic filters $\left\{G_{\lambda^{\prime }}\right\}$ leads to the continuous family of forcings $V[G_{\lambda^{\prime }}]$, as in Lemma 3. This breaks the global initial symmetry with the Hamiltonian $\hat{H}$ with the vanishing interaction part $H_{I}=0$ of the system and the observer (cf. [14] (Equations (5) and (6))). Then, it can be directly shown that $|\phi_{S}\left(\lambda^{\prime }\right)\rangle$ satisfies TDSE [14]:

$$\frac{i\partial }{\partial \lambda^{\prime }}|\phi_{S}\left(\lambda^{\prime }\right)\rangle =\hat{H}|\phi_{S}\left(\lambda^{\prime }\right)\rangle .$$

The parameter $\lambda^{\prime }$ corresponds to time *t* through $t=\lambda^{\prime }\hbar$. The family of forcings $V\to V^{B}/G_{\lambda^{\prime }}\to V\left[G_{\lambda^{\prime }}\right]$ triggers the breaking of the global symmetry (decoupling of $\phi_{S}\left(\lambda^{\prime }\right)$ in *V*) as above and determines the arrow of time,

$$\mathrm{for}\;\mathrm{any}\;\mathrm{two}\;\lambda_{1}^{\prime },\lambda_{2}^{\prime }\in R_{\geq 0}\lambda_{1}^{\prime }<\lambda_{2}^{\prime }\equiv V\left[G_{1}\right]\to V\left[G_{1}\right]\left[G_{2}\right]$$

according to Lemma 3 and the QM measure algebra *B* that guarantees the nontriviality of the above forcing extensions.

**Figure 1:**
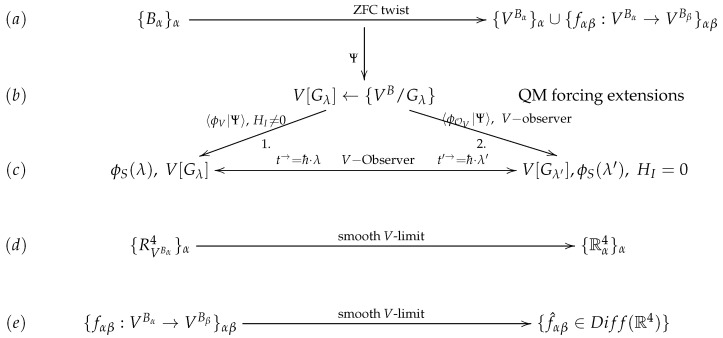
(**a**) indicates the ZFC twist that sends *B* to $V^{B}$ and $B_{\alpha }\to B_{\beta }$ to $V^{B_{\alpha }}\to V^{B_{\beta }}$. Then (**d**,**e**) show the result of taking the smooth limits for the above expressions in *V*. These smooth *V* limits result together in exotic smooth *R*^4^s in *V*. Path 1 (**b**,**c**) corresponds to the emergence of coordinate-like time through interactions with the environment *H_I_* ≠ 0. The forcing is in the background in (**b**). Path 2 indicates the emergence of time via forcing of the generic measurements of the *V*-observer. Here, *H_I_* = 0.

Forcing is also responsible for the arrow of time in the case of an interacting semiclassical environment. The *V*-based environment plays the role of the *V*-observer where the infinite dimensionality of the Hilbert spaces of Ψ and the environment leads to the nontrivial forcing extension and the arrow of time. As shown in [14], the derivation of time follows the interactions with the environment with arbitrary interacting Hamiltonian $H_{I}$, and this is also accompanied by the forcing extensions of models, as we discussed before (10). The dynamical processes then occur with respect to such a time variable. We present the arguments in a concise way in the diagram in Figure 1.

## 5. Discussion

The presented approach to the emergence of time in QM deserves and also requires further studies both in the formal mathematical part and in its justification in physics, especially in cosmology. However, it relies on well-established mathematics in the domain of axiomatic set theory like the forcing technique, and its application to physics is still a novelty which it is certainly worth pursuing in many directions. The ZFC twist of QM focuses on the set-theory content of QM rather than on quantum logic, but its performance opens new possibilities that widen our understanding substantially. This was the case for QM randomness, where certain sequences of QM outcomes negating Tsirelson’s conjecture were construed [5], and the approach to QG emerged [1]. The special feature underlying the construction via ZFC is the state of infinity when passing from QM to QFT, which enables the infinite number of degrees of freedom of the theories or the process of reaching the infinite dimension of Hilbert spaces to be seen from different angles [1,19,34].

The use of forcing and ZFC models is quite universal and can be applied to numerous physical theories and contexts. The emergence of the local-coordinate arrow of time presented here is based on random forcing due to the ZFC twist of QM. The interaction with the semiclassical environment, where time is already present, leads to its appearance in QM [14], with random forcing that induces the arrow of the flow of time. For an initial state of the universe which is purely quantum we needed continuum infinitely many forcing extensions determining the arrow of time and a kind of inflation without the semiclassical environment but solely based on QM. This is a quite strong result that also needs to be further analyzed formally. In particular, the continuum hypothesis and its independence from ZF axioms, which Cohen proved when he invented the forcing technique [35], can also become a valid part of the analysis. The formal possibility to reach cardinalities higher than c for $2^{\omega }$ can become an additional factor inducing the observed isotropy and homogeneity on large scales in the universe, thus leading to a ZFC-induced version of the cosmological principle. The assumption behind CP and certain possible deviations from CP [36,37] could find deeper explanations in the QM formalism with ZFC twist. In mathematics this is truism, in physics we think it can also be so. One such exemplification is probably the negation of Tsirelson’s conjecture on infinite-dimensional Hilbert spaces.

One could wonder whether the use of highly abstract constructions, such as higher cardinals and so on, can have anything to do with physical explanation and verification. Do we really need such abstract constructs? There are numerous researchers who state that physics is inherently finite, so this physics does not need any additional hyper-infinities. Time will show. Our understanding is such that the world as it functions is not just a simple addition of complications in a well-controlled computable process. Infinity can be reached step by step recursively, but it can also be present as a final ‘object’, without the possibility to decompose it into a collection of easy steps. Other possible examples of this kind are consciousness in physical systems and the physics behind the factor $III_{1}$ of Von Neumann algebras [38]. The irreducibility inherent in certain physical phenomena makes it impossible to predict when and where some abstract constructs will be necessary in the process of explaining the physical world.

Studies on the emergence of time in QM by ZFC forcing is the next step following the ZFC approach to randomness in QM [19] that followed the work on the formalization of physical theories [3].

Let us comment on the eventual experimental verification of some theoretical ideas on which this work is based. There are potentially two main pillars of such verification. One is a refined version of randomness in QM such that when grasped by finite quantum correlations, one could separate by the same token the special class of QM sequences negating the Tsirelson conjecture. This kind of randomness, called Solovay randomness, has been shown theoretically to negate the Tsirelson conjecture, which is known so far ‘only’ by a theoretical construction [28]. Solovay randomness is precisely based on the random Boolean algebra which has been extensively used in this paper. Eventual experimental verification of the negation of the Tsirelson conjecture would strongly favor the QM formalism built on quantum set theory and, as a consequence, the operational presence of random forcing in QM (seen in finitely many quantum correlations) [5]. Currently, indeed, a strong research effort is devoted to verifying from experiments the negation of the Tsirelson conjecture in QM [39].

The other class of eventual experimental insights into the approach in this paper is the problem of spacetime smoothness verified to very high energies or small lengths. Our approach predicts a kind of smooth phase transition in very high energies in the Planck era [40] rather than an emergence of a smooth spacetime from a discrete primordial phase. This means that the evolution of spacetime begins with the formation of an exotic smoothness on $R^{4}$ or eventually on $S^{4}$ and this exotic smoothness is, in particular, the source of the observed value of the cosmological constant [41]. Here, we have grouped certain formal reasons for creating such exotic smoothness directly from QM. This mechanism is based on forcing [2] and complements the emergence of the time arrow in QM. Thus, experimental investigations of the primordial beginning of the spacetime smoothness from quantum fluctuations could be helpful in the discrimination between the forcing-based scenario of the emergence of time in QM and some others [42]. A particularly important and general question would be to what extent inflation can be replaced by set-theory extensions of models. We want to systematically analyze this problem in a separate publication, also taking into account experimental bounds.

New techniques yield new possibilities, and old problems can gain quite surprising solutions. Distant from physics for years, deep results in formal set theory might become valid tools in the explanation of the physical world.

## Data Availability

No new data were created or analyzed in this study. Data sharing is not applicable to this article.
